# In Vitro and Ex Vivo Evaluation of Fluocinolone Acetonide–Acitretin-Coloaded Nanostructured Lipid Carriers for Topical Treatment of Psoriasis

**DOI:** 10.3390/gels8110746

**Published:** 2022-11-17

**Authors:** Hassan Raza, Shefaat Ullah Shah, Zakir Ali, Atif Ullah Khan, Irfa Basharat Rajput, Arshad Farid, Mohammed Al Mohaini, Abdulkhaliq J. Alsalman, Maitham A. Al Hawaj, Saima Mahmood, Abid Hussain, Kifayat Ullah Shah

**Affiliations:** 1 Department of Pharmacy, Faculty of Biological Sciences, Quaid-i-Aam University, Islamabad 45230, Pakistan; 2 Faculty of pharmacy, Gomal University, Dera Ismail Khan 29050, Pakistan; 3 Gomal Center of Biochemistry and Biotechnology, Gomal University, Dera Ismail Khan 29050, Pakistan; 4 Basic Sciences Department, College of Applied Medical Sciences, King Saud bin Abdulaziz University for Health Sciences, Alahsa 31982, Saudi Arabia; 5 King Abdullah International Medical Research Center, Alahsa 31982, Saudi Arabia; 6 Department of Clinical Pharmacy, Faculty of Pharmacy, Northern Border University, Rafha 91911, Saudi Arabia; 7 Department of Pharmacy Practice, College of Clinical Pharmacy, King Faisal University, Ahsa 31982, Saudi Arabia; 8 Department of Pharmacy, Faculty of Medical and Health Sciences, University of Poonch Rawalakot, Rawalakot 12350, Pakistan

**Keywords:** fluocinolone acetonide, acitretin, nanostructured lipid carrier, bioavailability, occlusive effect, entrapment efficiency

## Abstract

Psoriasis is chronic autoimmune disease that affects 2–5% of the global population. Fluocinolone acetonide (FLU) and acitretin (ACT) are widely used antipsoriatic drugs that belong to BCS classes II and IV, respectively. FLU exhibits side effects, such as skin irritation and a burning sensation. ACT also shows adverse effects, such as gingivitis, teratogenic effects and xerophthalmia. In the present study, topical nanostructured lipid carriers (NLCs) were fabricated to reduce the side effects and enhance the therapeutic efficacy. FLU–ACT-coloaded NLCs were prepared by the modified microemulsion method and optimized by the Box–Behnken model of Design Expert^®^ version 12. The optimization was based on the particle size (PS), zeta potential (ZP) and percentage of encapsulation efficiency (%EE). The physicochemical analyses were performed by TEM, FTIR, XRD and DSC to assess the morphology, chemical interactions between excipients, crystallinity and thermal behavior of the optimized FLU–ACT-coloaded NLCs. The FLU–ACT-coloaded NLCs were successfully loaded into gel and characterized appropriately. The dialysis bag method and Franz diffusion cells were used for the in vitro release and ex vivo permeation studies, respectively. The optimized FLU–ACT-coloaded NLCs had the desired particle size of 288.2 ± 2.3 nm, ZP of −34.2 ± 1.0 mV and %EE values of 81.6 ± 1.1% for ACT and 75 ± 1.3% for FLU. The TEM results confirmed the spherical morphology, while the FTIR results showed the absence of chemical interactions of any type among the ingredients of the FLU–ACT-coloaded NLCs. The XRD and DSC analyses confirmed the amorphous nature and thermal behavior. The in vitro study showed the sustained release of the FLU and ACT from the optimized FLU–ACT-coloaded NLCs and FLU–ACT-coloaded NLC gel compared with the FLU–ACT suspension and conventional gel. The ex vivo study confirmed the minimal permeation of both drugs from the FLU–ACT-coloaded NLC gel.

## 1. Introduction

Globally, 2–5% of the population is affected by psoriasis, and almost 2% of it is reported in the United States [1,2]. Psoriasis is a chronic autoimmune disease that is marked by red and itchy skin plaques [3]. It is triggered by hereditary, biological and environmental factors [4,5]. Presently, phototherapy, topical therapy and systemic therapies are crucial strategies for the management of psoriasis [6], even though dermal treatment is highly preferred for the moderate type of psoriasis [7]. Individuals with psoriasis using a compounded monotherapy have greater adherence (88%) than those who use two separate topical medications (51%) [8].

Fluocinolone acetonide (FLU) is a synthetic hydrocortisone derivative that is mainly used to treat skin inflammation and reduce itching [9]. FLU-blended NLCs and salicylic acid-augmented gel have been reported. Their clinical rewards for the management of psoriasis are assigned to their anti-inflammatory, immunosuppressive and antiproliferative properties [10]. FLU-loaded SLNs have also been reported [11]. However, serious cutaneous and systemic side effects are major concerns with the use of corticosteroids [12]

Acitretin (ACT) is a synthetic retinoid [13]. It is metabolite of vitamin A and has procured great interest due to its multiple physiological effects, such as the regulation of the epithelial cell growth and differentiation, sebum production and collagen synthesis [14]. It is the only FDA-approved drug used for the severe type of psoriasis [15]. Recently, ACT-loaded solid lipid nanoparticles (SLNs) have been reported [16].

Nanostructured lipid carriers (NLCs) are referred to as the advance form of solid lipid nanoparticles (SLNs), and they are composed of a mixture of solid and liquid lipids [5]. The consolidation of liquid and solid lipids produces great flaws in the crystal lattice and leaves enough space for the settlement of the drugs [17]. They have been introduced in order to overcome the restrictions of SLNs (i.e., drug-payload and drug-leaking phenomena) [18]. NLCs have also been reported to offer several advantages over conventional topical products owing to their ability to prolong the drug release, minimize skin irritation and protect the drug from possible degradation. Additionally, the high surface area of the particles certifies excellent contact with the inflamed skin [19]. They also have enhanced solubility and minimal dose-dependent side effects [20]. Furthermore, NLCs assist in the more efficient transfer of drugs [21,22].

ACT belongs to BCS class IV, having low bioavailability (60%). It is selective for the retinoid acid receptor (RAR) [23]. The common adverse effects of ACT are dry lips, gingivitis and xerophthalmia [24]. FLU belongs to BCS II, and it has severe topical dose-dependent side effects, such as skin irritation, a burning sensation and steroidal acne [25].

To address the above problems associated with FLU and ACT, they were encapsulated in NLCs. The colloidal system was prepared by mixing solid and liquid lipids through the modified microemulsion method. The incorporation of FLU–ACT in NLCs is a superior choice, as they release the drug in a sustained manner with minimal side effects.

## 2. Results and Discussion

### 2.1. Preparation and Optimization of FLU–ACT-Coloaded NLCs

**Figure 1:**
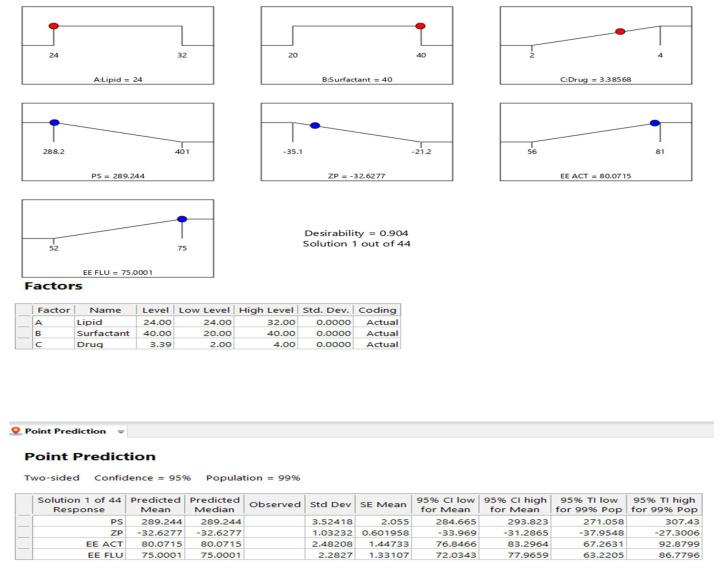
Desirability and point prediction data provided by Design Expert^®^ for the selection of the optimized formulation.

The FLU–ACT-coloaded NLCs were prepared via the microemulsion method. The microemulsion method was chosen due to its simple processing method and low-energy-input requirement. NLCs with a monodispersed nature can be fabricated via this method [26]. Miltefosine-loaded NLCs with high entrapment and a stable nature have been prepared through this technique [27]. The optimization of the FLU–ACT-coloaded NLCs was carried out through the Box–Behnken model of Design Expert^®^. The concentrations of the drugs, solid lipid and surfactants were varied, and their responses were assessed based on the particle size (PS), zeta potential (ZP) and encapsulation efficiency (%EE). Table 1 shows the effects of these variables on the PS, ZP and %EE of ACT and FLU. Figure 1 shows the results of the point prediction and desirability, on the basis of which Formulation 3 was selected as the optimized formulation, with a PS of 288.2 ± 2.3 nm, ZP of −34.2 ± 1.0 mV and %EE values of 81.6 ± 1.1% for ACT and 75 ± 1.3% for FLU.

### 2.2. Characterization of FLU–ACT–Coloaded NLCs

#### 2.2.1. Effects of Variables on Particle Size

Significant effects were shown by all the independent variables on the particle size with a p-value of < 0.001. The lack of fit was nonsignificant (0.5846), and the difference between the adjusted R^2^ (0.9897) and predicted R^2^ (0.9833) was less than 0.2. The high value of the F (417.87) indicated that there was only a 0.01% chance that an F-value this large could occur due to noise.

**Figure 2:**
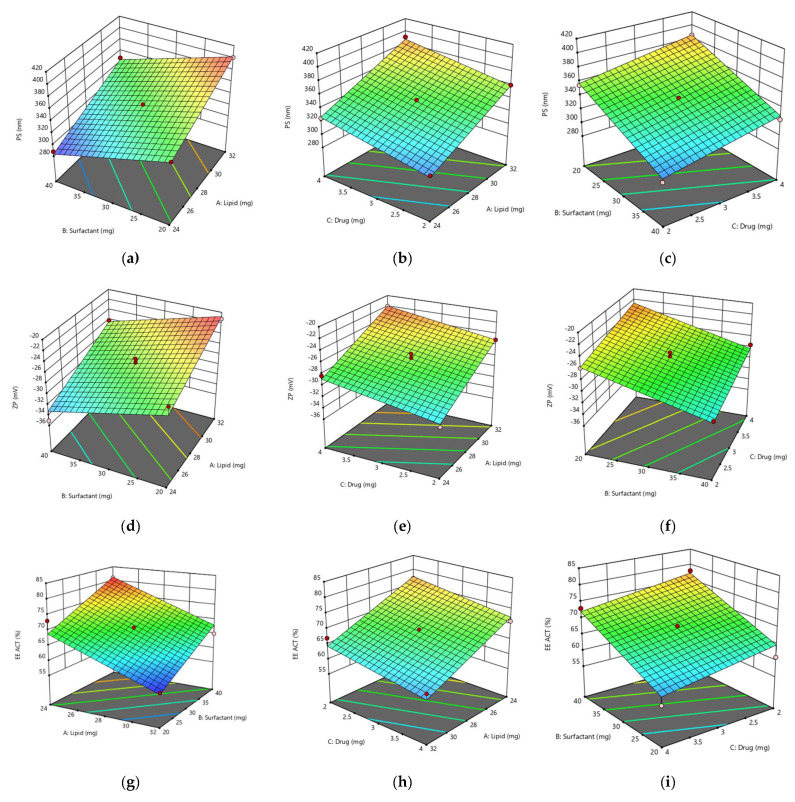

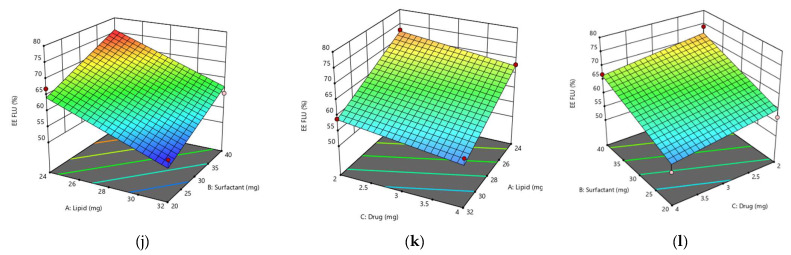
3D response surface graphs: effects of lipids, surfactants and drugs on particle size (**a**–**c**); zeta potential (**d**–**f**): % entrapment efficiency of acitretin (**g**–**i**): % entrapment efficiency of fluocinolone (**j**–**l**).

Figure 2a–c shows the effects of the ACT and FLU, lipid and surfactant concentrations on the PS. It was observed that whenever the concentration of solid lipid was reduced, the PS decreased, and when the drug concentration was increased from 2 mg to 4 mg, the PS increased. The explanation for the decrease in the PS is that, whenever the solid lipid concentration decreases, the liquid lipid concentration ultimately rises. Increasing the amount of liquid lipid causes a reduction in the viscosity, and as a result, the interfacial tension decreases, which contributes to the reduction in the PS [28]. The explanation for the increase in the PS with the higher drug content could be the increase in the viscosity of the molten lipid. A high viscosity makes it difficult to disperse two phases, which results in larger particle sizes. Sanad et al. observed a similar outcome for oxybenzone-loaded NLCs [29]. When the concentration of the surfactant was increased, a reduction in the PS was observed. This decrease in the PS could be due to the reduction in the interfacial tension, which contributes to the easier dispersion of the two phases with a smaller particle size. Yu et al. showed similar results for sirolimus NLCs [30].

#### 2.2.2. Effects of Variables on Zeta Potential

Significant effects were shown by all the independent variables on the ZP with a *p*-value of < 0.001. The lack of fit was nonsignificant (0.3437), and the difference between the adjusted R^2^ (0.9261) and predicted R^2^ (0.8811) was less than 0.2. The high value of the F (55.34) indicated that there was only a 0.01% chance that an F-value this large could occur due to noise.

The ZP value indicates the stability of nanoparticles. Nanoparticles with a ZPs larger than ± 25 mV are said to be stable. The effects of the solid lipid, ACT, FLU and surfactant concentrations on the ZP value are shown in Figure 2d–f. Because of the reduction in the PS, which could increase the charge density on the surfaces of nanoparticles, a drop in the solid lipid concentration leads to an increase in the ZP value towards the negative side [31]. As the surfactant concentration is increased, the ZP also increases because of the reduction in the PS, which subsequently leads to an increased charge density on the surfaces of nanoparticles [31]. Increased concentrations of FLU and ACT could result in a decrease in the ZP value due to the increase in the PS, which leads to a reduction in the negative ZP value.

#### 2.2.3. Effect of Variables on Entrapment Efficiency

Significant effects were shown by all the independent variables on the %EE of the ACT and FLU with a *p*-value of < 0.001. The lack of fit was nonsignificant (0.3437 for ACT and 0.4363 for FLU), and the difference between the adjusted R^2^ (0.9261) and predicted R^2^ (0.8811) for the ACT, and between the adjusted R^2^ (0.9105) and predicted R^2^ (0.8570) for the FLU, was less than 0.2. The high values of the F (55.34 (ACT) and 45.05 (FLU)) indicated that there was only a 0.01% chance that an F-value this large could occur due to noise.

The influences of the solid lipid and drug concentrations on the %EE of the ACT and FLU are depicted in Figure 2g–l. When the concentration of solid lipid was decreased, an increasing trend was observed in the %EE. The drop in the solid lipid concentration eventually leads to an increase in the liquid lipid concentration, which boosts the drug solubility in the NLC matrix and may cause crystal order disruption, which results in many defects in the NLC matrix and therefore increases the %EE. The findings were consistent with those of Sanad et al. [29]. There is direct relationship between solid lipid and the %EE, and the entrapment was enhanced by increasing the lipid concentration. The possible reason might be the greater capacity of lipids to accommodate more drugs in the lipid layers [32]. Because the amount of lipid available to accept increased drug concentrations could be insufficient, a decreasing trend in the %EE was observed with the increasing drug concentration beyond a certain limit. The findings were comparable to those of Din et al. [33]. With the increasing surfactant content, an increasing trend in the %EE was seen due to the decreased interfacial tension between the lipid phase and the drug. The findings were consistent with those of Bahari et al. [34].

### 2.3. Characterization of FLU–ACT–Coloaded NLCs

#### 2.3.1. Particle Size, Zeta Potential, Polydispersity Index (PDI) and Entrapment Efficiency

**Figure 3:**
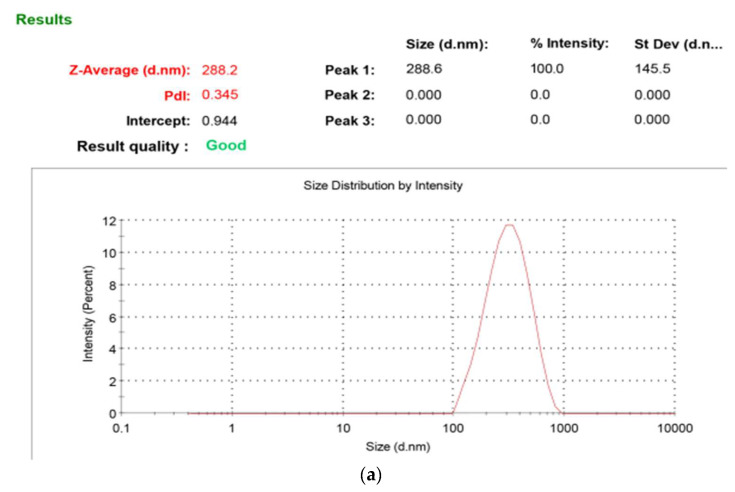

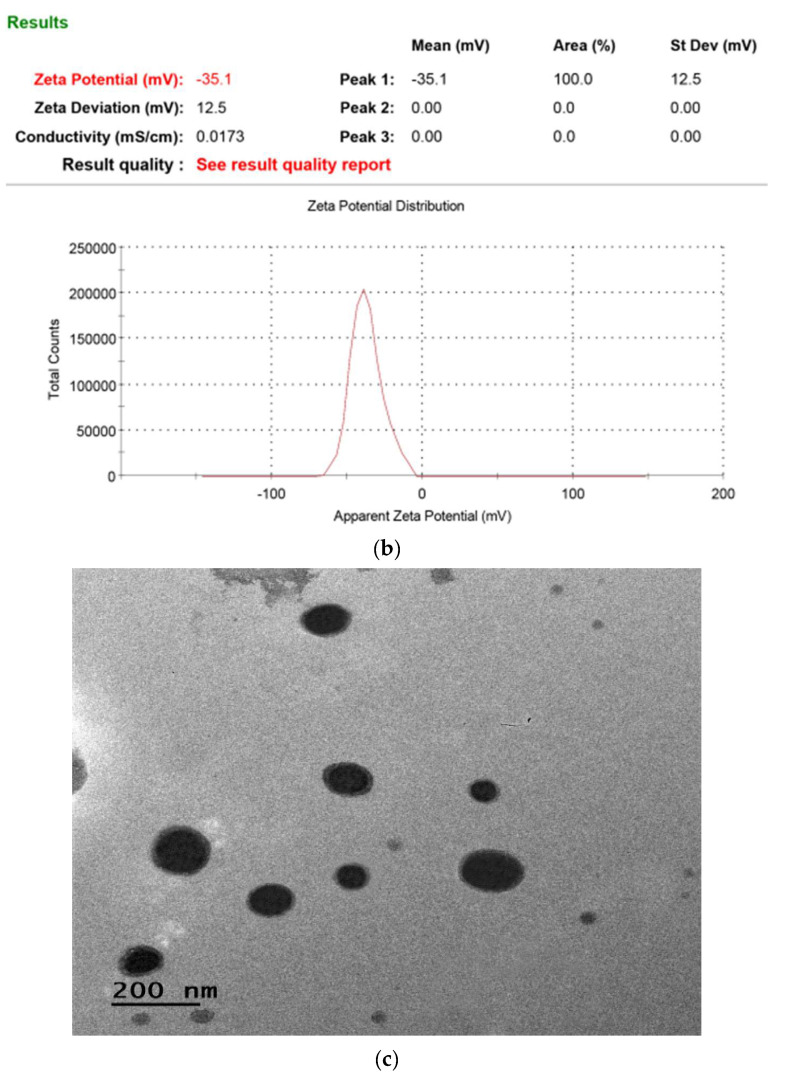
Particle characterization: (**a**) particle size and PDI analysis; (**b**) zeta potential analysis; (**c**) TEM analysis.

The nanosize of the formulation was confirmed by the results of the dynamic-light-scattering analysis. Figure 3 depicts the results of the optimized formulation. The optimized formulation has a PS of 288.2 ± 2.3 nm, PDI of 0.345 ± 0.005 (Figure 3a) and ZP of −34.2 ± 1.0 mV (Figure 3b), indicating the nanosize range and stable and homogeneous nature of the optimized FLU–ACT-coloaded NLCs. The %EE values of the FLU–ACT-coloaded NLCs was 81.6 ± 1.1% for ACT and 75 ± 1.3% for FLU.

#### 2.3.2. Transmission Electron Microscope (TEM) Analysis

A TEM analysis was performed to assess the surface morphology of the optimized FLU–ACT-coloaded NLCs. The results shown in Figure 3c, demonstrated that all the particles were spherical, homogeneous, well segregated and in the nanosized range, thus complying with the results of the zeta analysis [35].

#### 2.3.3. Powder X-ray Diffractometry (PXRD) Analysis

**Figure 4:**
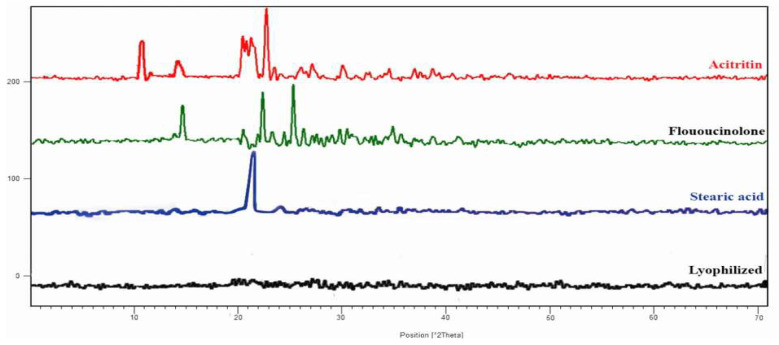
XRD analysis of acitretin, fluocinolone, stearic acid and lyophilized optimized FLU–ACT-coloaded NLCs.

A PXRD analysis was performed to determine the changes in the polymorphic nature of the ACT, FLU and stearic acid. Characteristic crystalline peaks at 2θ angles of 11°, 15°, 21°, 23° and 24° were observed in the diffractogram of the ACT, owing to its higher crystalline nature [36]. Similarly, a principal peak at 21° was observed in the diffractogram of the stearic acid [37]. Moreover, the FLU executed sharp characteristic crystalline peaks at 15°, 19°, 22°, 24° and 25° [38], whereas such peaks disappeared in the case of the NLCs, which may be due to the conversion of the stearic acid, ACT and FLU from a crystalline form to an amorphous form, and the successful incorporation of both ACT and FLU into the NLCs (Figure 4).

#### 2.3.4. Differential Scanning Calorimetry (DSC) Analysis

**Figure 5:**
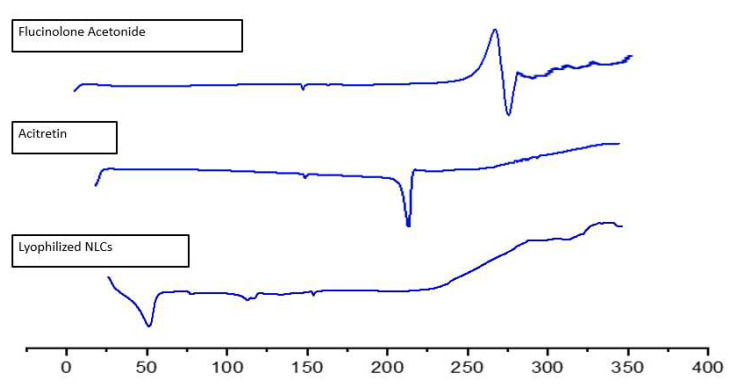
DSC analyses of fluocinolone, acitretin and optimized FLU–ACT-coloaded NLCs.

A DSC analysis was used to assess the melting behavior of the ACT, FLU and FLU–ACT-coloaded NLCs (Figure 5). The pure ACT displayed a pronounced endothermic peak at around 220 °C, corresponding to its melting point. The pure FLU showed a strong endothermic event at 265 °C that was related to its melting temperature, indicating the crystalline structure of the pure drugs. The peaks for the FLU and ACT disappeared in the thermal curve of the lyophilized FLU–ACT-coloaded NLCs. These results suggest the conversion from the crystalline state of the FLU and ACT to an amorphous one in the FLU–ACT-coloaded NLCs [11].

#### 2.3.5. Fourier-Transform Infrared Spectroscopy (FTIR) Analysis

**Figure 6:**
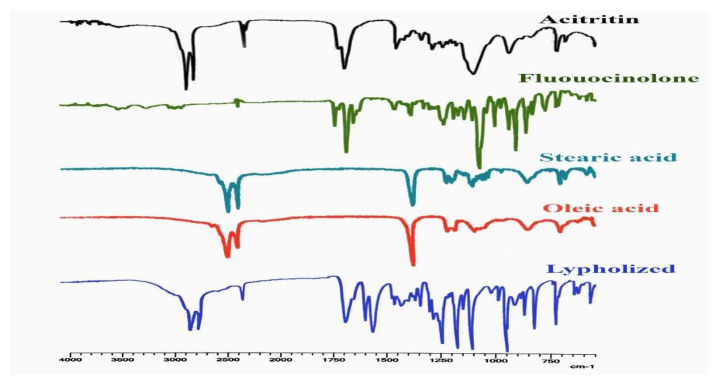
FTIR analysis of fluocinolone, acitretin, stearic acid, oleic acid and lyophilized FLU–ACT-coloaded NLCs.

An FTIR analysis was conducted to confirm the absence of chemical interactions among the ingredients of the formulation. The FTIR spectrum of the FLU showed principal peaks at 2902 cm^−1^, 1640 cm^−1^ and 1728 cm^−1^, representing C–H, C=C and C=O group stretching, respectively [39]. Similarly, the ACT showed characterized peaks around 1700–1500 and 1300–1100 cm^−1^ [40]. The oleic acid showed principal peaks at 2922 cm^−1^, 2853 cm^−1^, 1708 cm^−1^, 1412 cm^−1^, 1285 cm^−1^ and 935 cm^−1^ that indicated asymmetric CH_2_ stretching, symmetric CH_2_ stretching, C=O stretching, OH stretching in plane, OH stretching out plane and C–O stretching, respectively [41]. The spectrum of the stearic acid showed principal peaks at 2955 cm^−1^, 2847 cm^−1^, 1697 cm^−1^, 1294 cm^−1^ and 721 cm^−1^, demonstrating OH, CH and C=O stretching [42]. The FTIR spectra of the FLU–ACT-coloaded NLCs showed all the corresponding peaks, demonstrating no chemical interactions between the different components of the FLU–ACT-coloaded NLCs (Figure 6).

#### 2.3.6. Preparation and Characterization of FLU–ACT-Coloaded NLC Gel

The optimized FLU–ACT-coloaded NLCs were successfully loaded into the Carbopol 934-based gel. The prepared gel had a slightly green-yellow appearance due to the addition of ACT. The gel was free of gritty particles and lumps, showing the homogeneous nature [43]. It was previously reported that both acidic and basic pH values may cause skin irritation, and so the pH of the gel was adjusted to 7 via the addition of tri-ethanol amine [44]. A rheological study showed the non-Newtonian behavior of the gel. The evaluation of the rheological behavior of the optimized FLU–ACT-coloaded NLC gel was carried out to demonstrate its application, adhesion and retention on the skin surface. In the case of the topical formulation, the non-Newtonian behavior is favorable as it allows for the maximum coverage of the skin area [45].

#### 2.3.7. Drug Content and Spreadability

The maintenance of the dosing homogeneity and the uniform application of the gel on the skin surface are important parameters to achieve a better therapeutic effect and patient compliance. The spreadability of the gel has a positive relation with its in vivo therapeutic efficacy. Earlier reports demonstrated better therapeutic effects with a more uniformly applied gel [33]. The optimized FLU–ACT-coloaded NLC gel exhibited a spreadability of 265 ± 3.2%, demonstrating the adequate spreadability over the skin surface. The drug content was 95.1 ± 1.2% and 91.3 ± 0.6% for the ACT and FLU, respectively. These results demonstrated the good distribution of the FLU–ACT-coloaded NLCs within the gel. The percentage of both drugs for the topical application was in the range that is reported in the literature (90–110% range, according to the regulatory requirements) [46].

#### 2.3.8. Skin Irritation Study

**Figure 7:**
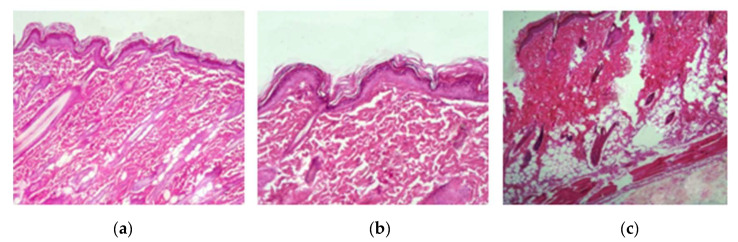
Histopathological analysis of: (**a**) positive control; (**b**) FLU–ACT–coloaded NLC treatment; (**c**) negative control.

The safety and nonirritant nature of the FLU–ACT-coloaded NLC gel was evaluated via a skin irritation study. The Draize scoring method was used to evaluate the cutaneous irritation (Table 2). Skin treated with the FLU–ACT-coloaded NLC gel was evaluated and compared with 0.8% formalin-treated skin (negative control) and untreated skin (positive control). The primary dermal irritation (PDI) score was calculated on the basis of the presence or absence of edema and/or erythema. The (PDII) values of the FLU–ACT-coloaded NLC gel and positive control were 0.33 and 0.00, respectively (calculated from PDI values), while the PDII value for the negative control group was 4.66, which was significantly higher compared with the positive control group and the group treated with the FLU–ACT-coloaded NLC gel. The results of the in vivo skin irritation investigation were backed up by histopathological findings, as shown in (Figure 7). The histological results of the skin irritation study revealed that the skin tissues were intact in the positive control group and the group treated with the FLU–ACT-coloaded NLC gel, while the 0.8% formalin-treated group showed the infiltration of the inflammatory cells, along with loose textured collagen and dermal damage.

#### 2.3.9. In Vitro Drug Release

**Figure 8:**
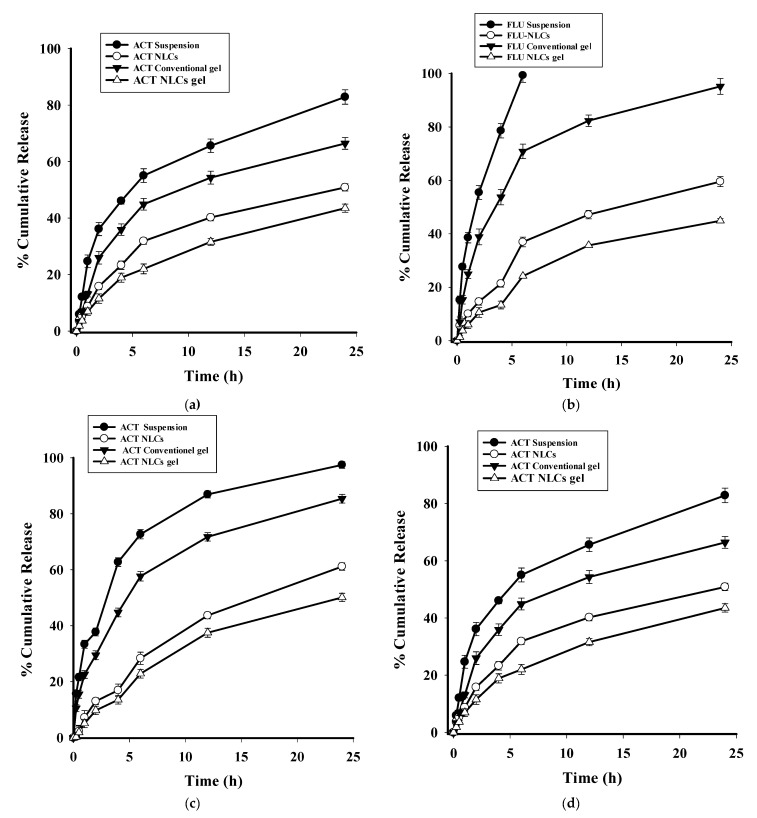
In vitro release profiles of fluocinolone at (**a**) pH of 5.5 and (**b**) pH of 7.4, and acitretin at (**c**) pH of 5.5 and (**d**) pH of 7.4.

The in vitro release study was carried out at two different pH values: a pH of 5.5, corresponding to the skin, and a pH of 7.4, corresponding to the blood pH. At a pH of 5.5, the FLU suspension showed 33.7% release in the initial 2 h, and complete release in 24 h, while from the FLU–ACT-coloaded NLCs, 19.3% and 68.9% of the FLU was released in the initial 2 h and 24 h, respectively. At this particular pH, the FLU conventional gel showed 26.4% and 84.0% release in the initial 2 h and 24 h, while the FLU–ACT-coloaded NLC gel showed 13.2% and 58.3% release in 2 h and 24 h, respectively (Figure 8a). At a pH of 7.4, the FLU suspension showed 59.4% release in the initial 2 h, and 99.3% release in 6 h, while from the FLU–ACT-coloaded NLCs, 14.6% and 59.54% of the FLU was released in the initial 2 h and 24 h, respectively. At this particular pH, the FLU conventional gel showed 38.8% and 95.17% release in the initial 2 h and 24 h, respectively, while the FLU–ACT-coloaded NLC gel showed 10.55% and 44.8% release in 2 h and 24 h, respectively (Figure 8b). At a pH of 5.5, the ACT suspension showed 37.74% release in the initial 2 h, and 97.4% release in 24 h, while from the FLU–ACT-coloaded NLCs, 13.0% and 61.15% of the ACT was released in the initial 2 h and 24 h, respectively. The ACT conventional gel exhibited 29.45% and 85.4% release in the initial 2 h and 24 h, respectively, while the FLU–ACT-coloaded NLC gel demonstrated 9.7% and 50.1% release in 2 h and 24 h, respectively (Figure 8c). At a pH of 7.4, the ACT suspension showed 36.11% release in the initial 2 h, and 86.8% release in 24 h, while from the FLU–ACT-coloaded NLCs, 15.8% and 50.8% of the ACT was released in the initial 2 h and 24 h, respectively. The ACT conventional gel showed 25.9% and 66.4% release in the initial 2 h and 24 h, respectively, while the FLU–ACT-coloaded NLC gel showed 11.48% and 43.49% release in 2 h and 24 h, respectively (Figure 8d). From the abovementioned release patterns, it can be concluded that the FLU–ACT-coloaded NLCs and FLU–ACT-coloaded NLC gel provide sustained release compared with the FLU–ACT suspension and FLU–ACT conventional gel at both the mentioned pH mediums. The sustained release behavior for both drugs might be attributed to their incorporation into nanoparticles, and might also be due to the stability imparted by Tween 80 via the formation of a uniform layer around the nanoparticles [47]. Another important factor that plays a role in sustained release is the presence of the additional barrier to the release provided by the gel matrix.

Different kinetic models were applied to the in vitro profiles of both FLU and ACT (Table 3), and the results suggest that the release of both FLU and ACT from the FLU–ACT-coloaded NLCs followed the Korsmeyer–Peppas model (R^2^ = 0.9770) for FLU, and R^2^ = 0.9850 for ACT (*n* = 0.53). The “*n*” value is smaller than 0.89; thus, the drugs followed non-Fickian diffusion, which is determined by the following equation:

Mt/M∞ = Kkp × t*n*

where Kkp is the release constant; Mt/M∞ is the portion of drug released in time (t); *n* is the diffusion exponent. The *n* represents the mechanism of drug release: if *n* < 0.45 shows Ficks law, then 0.45 < *n* < 0.89 shows non-Fickian diffusion [48].

#### 2.3.10. Ex Vivo Permeability Study

**Figure 9:**
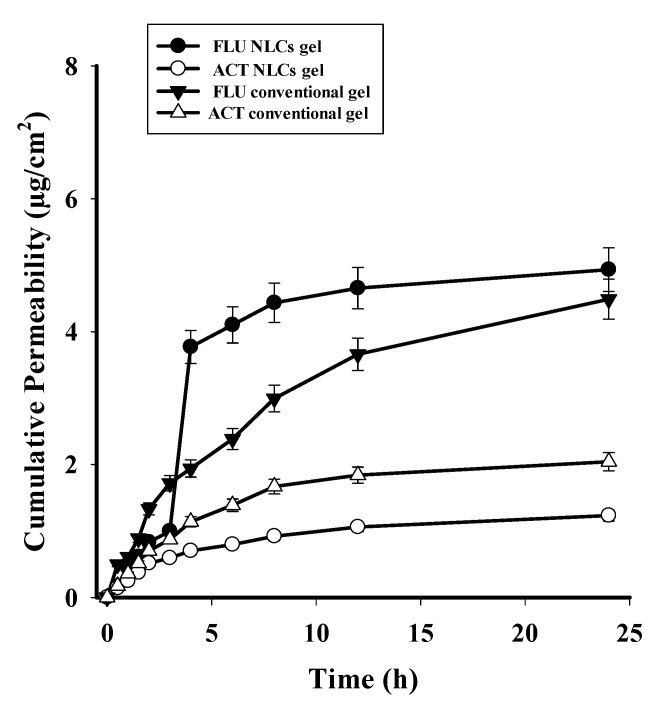
Ex vivo permeability data of FLU–ACT-coloaded NLC gel and FLU–ACT conventional gel.

The permeability study showed that the cumulative concentration of the drugs from the formulation gel and F conventional gel permeated/unit area of the skin (Figure 9). The ex vivo permeation data showed less permeation of the medication through the rat skin. In the case of the FLU–ACT-coloaded NLC gel, 5.05 µg/cm^2^ of the drug was permeated, while from the FLU conventional gel, 4.33 µg/cm^2^ drug was permeated after 24 h. From the ACT conventional gel, 1.24 µg/cm^2^ of the drug was permeated, while from the FLU–ACT-coloaded NLC gel, 2.02 µg/cm^2^ of the drug was permeated after 24 h. The permeation graph was plotted against time (h).

#### 2.3.11. Skin Deposition Study

The deposition study showed that almost 21.01% of the FLU and 18.89% of the ACT was retained from the FLU–ACT-coloaded NLC gel, while only 4.02% of the FLU and 3.13% of the ACT was deposited from the FLU–ACT conventional gel (Table 4).

#### 2.3.12. Stability Study

A stability study was conducted to evaluate its effect on the storage condition of the optimized FLU–ACT-coloaded NLCs for a period of 6 months, in accordance with the ICH guidelines (Q1A (R^2^)). The optimized FLU–ACT-coloaded NLCs were tested for their integrity and stability at two different temperatures: T_1_ = 25 ± 2 °C at 60% RH ± 5% RH (accelerated storage condition for drug substances to be stored at refrigerator temperature), and T_2_ = 40 ± 2 °C at 75% RH ± 5% RH (accelerated storage condition for general substances) [49]. The PS, ZP, PDI and %EE (FLU, ACT) of the freshly prepared formulation were 288.2 ± 2.3, −34.2 ± 1.0, 0.347 ± 0.002, 75.6 ± 1.3 and 81.6 ± 1.1, respectively. At T_1_, after a period of 6 months, the PS, ZP, PDI and %EE (FLU, ACT) were 292.3 ± 1.5 nm, −30.7 ± 1.0 mV, 0.349 ± 0.04, 68.3 ± 1.0% and 76.1 ± 1.9%, respectively. Similarly, at 40 °C, after 6 months, the PS, ZP, PDI and %EE (FLU, ACT) were 292.8 ± 3.2 nm, −31.0 ± 1.7 mV, 0.349 ± 0.005, 66 ± 1.5% and 75.9 ± 1.1%, respectively. No significant changes in the parameters were observed after 6 months of storage, which thus proved that the formulation was stable at both room temperature and refrigerator temperature (Table 5).

## 3. Conclusions

NLCs were optimized by Design Expert^®^ version 12 using the Box–Behnken model. The optimized formulation had a PS of 288.2 ± 2.3 nm, ZP of −34.2 ± 1.0 mV and %EE values of 81.6 ± 1.1% for ACT and 75 ± 1.3% for FLU. The TEM analysis of the optimized FLU–ACT-coloaded NLCs showed oval or nearly spherical shapes, with a PS greater than 200 nm. The XRD analysis showed characteristic crystalline peaks for both drugs and the lipids, while these peaks were absent in the lyophilized NLCs, thus providing evidence that the FLU–ACT-coloaded NLCs were successfully transformed into the amorphous form. The DSC thermogram showed that both drugs had sharp endothermic peaks, while the peaks were diminished in the lyophilized formulation, thus indicating the conversion of the crystalline form to an amorphous one. The FTIR analysis revealed the absence of chemical interactions between the FLU–ACT-coloaded NLC ingredients. The Carbapol-934 based gel showed the non-Newtonian behavior of the gel. The skin irritation analysis confirmed the suitability of the gel for topical application. The in vitro release study confirmed the sustained release pattern of both drugs from the nanoformulation. The ex vivo permeation results showed a more minimal permeation for the FLU–ACT-coloaded NLC gel than the FLU–ACT conventional gel; however, the skin deposition of the NLC formulation was higher compared with the raw gel, which confirmed the presence of the NLCs in the deeper dermal layers. This might ultimately enhance the antipsoriatic activity of both ACT and FLU drugs. Thus, it can be concluded that the NLCs were suitable carriers for the topical delivery of ACT and FLU.

## 4. Materials and Methods

### 4.1. Materials

The stearic acid, oleic acid, absolute ethanol, dimethylformamide (DMF), Tween 80, Span 80, Carbopol 934, dialysis membrane tubes (12–14 kDa), disodium hydrogen phosphate, sodium chloride and sodium hydroxide were purchased from Sigma Aldrich (Islamabad, Pakistan). The fluocinolone acetonide was kindly gifted by Shaigan Pharmaceuticals (Rawalpindi, Pakistan). The acitretin was kindly gifted by Bio Labs Pharmaceuticals (Islamabad, Pakistan). Pure analytical grade reagents were used in this study.

### 4.2. Preparation of FLU–ACT-Coloaded NLCs

To prepare the FLU–ACT-coloaded NLCs, the microemulsion method was used with some modifications. In this method, the oily phase containing the stearic acid, oleic acid, drugs (FLU–ACT) and Span 80 were heated up to 80 °C on a hot plate (MS-H280-PRO, SCILOGEX, USA). Meanwhile, the aqueous phase containing a surfactant (Tween 80) was heated to the same temperature on a hot plate. The aqueous phase was then dispersed slowly in the molten oily phase and mixed on a magnetic stirrer at 1000 rpm for 1 h. The obtained o/w microemulsion was then homogenized at 10,000 rpm for 15 min via a homogenizer (D-91126, Heidolph, Germany). Finally, the resulting o/w microemulsion was dispersed in ice-cold water (2–3 °C) at a ratio of 1:9 (nanoemulsion:water) to obtain the FLU–ACT-coloaded NLCs [50].

### 4.3. Optimization of FLU–ACT-Coloaded NLCs

FLU–ACT NLCs were optimized via Design Expert^®^ version 12 using the Box–Behnken model. The concentrations of solid lipid (stearic acid), liquid lipid (oleic acid), drugs (FLU–ACT) and surfactants (Tween 80, Span 80) were varied, and the effects of these variations were observed on the PS, ZP and %EE. The effects of these variations on the properties of the different formulations are listed in Table 1 [51].

### 4.4. Characterization of FLU–ACT NLCs

#### Particle Size, Zeta Potential and Polydispersity Index

The PS, ZP and PDI of the FLU–ACT-coloaded NLCs were determined using a zeta sizer (ZS90) equipped with a He–Ne laser that works at a wavelength of 635 nm. All the measurements were carried out at a fixed light incidence angle (90) and 25 °C with software version 6.34 (Malvern Instruments, Worcestershire, UK). Prior to analysis, the samples were properly diluted by dispersing 10 µL of the FLU–ACT-coloaded NLCs in 1 mL of distilled water, and finally the samples were vertexed for 1 min [52].

### 4.5. Entrapment Efficiency (%EE) of FLU–ACT-Coloaded NLCs

The %EE was calculated by the indirect method. Briefly, 2 mL of FLU–ACT-coloaded NLCs was centrifuged at 10,000 rpm at 4 °C for 1.5 h via centrifuge (Herml labortechnik, Z-216 MK, Wehingen, Germany). After centrifugation, the supernatant was collected, and the amount of free drug was calculated via a spectrophotometer (HALO DB-20. UV-VIS Double Beam Spectrophotometer) at 238 nm and 354 nm for the FLU and ACT, respectively. The following equation was used to calculate the %EE values of the FLU and ACT:

$$\%\mathrm{EE}=\frac{Wt-W_{f}}{Wt}\;\times \;100$$

where *Wt* is the total drug concentration, and *W_f_* is the free drug concentration [53].

#### 4.5.1. Transmission Electron Microscopy (TEM)

The surface morphology of the FLU–ACT-coloaded NLCs was analyzed by TEM analysis (model: JEM 2100; energy: 200 KV; manufacturer: JEOL, Tokyo, Japan). A drop of diluted sample was placed on the surface of a carbon-coated copper grid and stained for 30 s with a drop of 2% (*w*/*w*) phosphotungstic acid aqueous solution (negative stain). Filter paper was used to remove any excess staining solution. For the analysis, a thin aqueous coating was left on the copper grid’s surface [11].

#### 4.5.2. Powder X-ray Diffractometry (PXRD)

XRD analysis was performed to survey the crystal lattice and amorphous behavior of the FLU–ACT-coloaded NLCs. XRD analyses of pure FLU, ACT, stearic acid and FLU–ACT-coloaded NLCs were studied by powder X-ray diffractometer (JDX 3532, JEOL, Tokyo, Japan). Samples were positioned in sample stage and scanned from 2 h to 60 h with an operating voltage of 40 kV and a current of 30 mA [54].

#### 4.5.3. Fourier-Transform Infrared Spectroscopy (FTIR)

The stability, solubility and chemical composition of a drug substance can be altered if there is any sort of chemical interaction between the formulation ingredients. An FTIR analysis of the FLU, ACT, stearic acid, physical mixture and lyophilized FLU–ACT-coloaded NLCs was conducted to ensure the absence of chemical interactions between the drugs and excipients. Prior to analysis, the samples were carefully pulverized and combined with potassium bromide. The discs were then used for FTIR spectra in a range from 4000 to 400 cm^−1^ [55].

#### 4.5.4. Diffraction Scanning Calorimetry (DSC)

The DSC thermogram was used to evaluate the thermal behavior of the FLU, ACT and lyophilized FLU–ACT-coloaded NLCs. Samples weighing approximately 5 mg were hermetically sealed in aluminum pans, covered with a lid and analyzed by DSC 822e (Mettler-Toledo International Inc., Columbus, OH, USA) at a temperature range of 0–350 °C at a rate of 10 °C/min [56].

### 4.6. In Vitro Release Study

The in vitro release behavior of the optimized FLU–ACT-coloaded NLCs, FLU–ACT suspension, FLU–ACT-coloaded NLC gel and FLU–ACT conventional gel was analyzed at two different pH values (5.5 and 7.4), corresponding to the skin and blood pH, respectively. The dialysis tube method was utilized for the conduction of the in vitro release study. The FLU–ACT-coloaded NLCs, FLU–ACT suspension, FLU–ACT-coloaded NLC gel and FLU–ACT conventional gel were taken in equal amounts (3 mg) and were filled in individual dialysis bags and placed in beakers containing 50 mL of the respective buffer medium. The complete wetting of the dialysis bags was achieved by immersing them in the respective buffers for a period of 30 min. The beakers containing dialysis bags were then placed in a shaker bath already maintained at a temperature of 37 ± 1 °C and shaking speed of 75 rpm. At a predetermined time interval (0.25, 0.5, 1, 2, 4, 6, 12 and 24 h), 1 mL of sample was collected, and the same amount of fresh buffer was added to maintain the sink condition. The amount of drug in each collected sample was analyzed spectrophotometrically at specific wavelengths for the FLU and ACT [57].

### 4.7. Preparation of Gel

To make the FLU–ACT-coloaded NLC gel applicable for topical use, the NLCs were loaded into Carbopol 934-based gel. A transparent dispersion was obtained by the slow addition of Carbopol 934 (1%) with continuous stirring into distilled water. Complete swelling and hydration were achieved via maintaining the Carbopol 934 dispersion overnight. Two–three drops of triethanolamine were added to neutralize the acidic pH of the Carbopol 934 dispersion. At the end, the prepared optimized FLU–ACT-coloaded NLCs were incorporated with 1 h of continuous stirring [58].

#### 4.7.1. Gel Characterization

##### Homogeneity and pH Determination

Visual inspection for visible particles, bubbles, lumps, color and transparency was used to determine the homogeneity of the gel [58]. The pH is an important consideration for formulations intended to be applied on the skin to ensure the nonirritant nature of the gel. A digital pH meter was used for the determination of the gel pH [59].

#### 4.7.2. Drug Content

Ethanol and DMF (100 mL) were used to dissolve one gram of FLU–ACT-coloaded NLC gel separately. After that, the prepared solution was sonicated, filtered and the drug content was determined using a UV–visible spectrophotometer [58].

#### 4.7.3. Rheological Study

The rheological study of the gel was carried out by using a rheometer (Brookfield). The viscosity was measured at different rpm values. The relation was determined between the viscosity and shear rate to evaluate the gel behavior [60].

#### 4.7.4. Spreadability Study

The glass slide method was utilized for the evaluation of the spreadability percentage of the FLU–ACT-coloaded NLC gel. A one-centimeter circle was marked on a slide, and 0.5 g gel was placed in the circle. A second slide was placed for covering the gel-containing slide, and a weight of 500 g was maintained over it for 5 min. After the removal of the weight, the increase in the gel diameter was measured, and the spreadability was calculated via the following equation:

$$Si=d^{2}\times \frac{\pi }{4}$$

where *Si* is the spreadability index, and *d* is the diameter (in mm) of the spread area [61].

### 4.8. Skin Irritation Study

A rat skin irritation study was conducted to determine the safety of the topical formulation on the skin. All rats had their back hair shaved, and they were placed into three groups. Group I was given formalin (0.8%), Group II was given FLU–ACT-coloaded NLC gel and Group III was given no treatment. For 24 h after applying the appropriate formulation, the rat skin was examined for erythema and edema. The primary dermal irritation index (PDII) was calculated using the Draize scoring method. The PDII scale ranges from 0 to 0.4, which show no irritation, and > 0.5–1.9, 2–4.9 and > 5 indicate mild, moderate and severe irritation, respectively. After 24 h, the rats were sacrificed via spinal dislocation, and the affected skin area was surgically excised and preserved in 10% formalin. Finally, slides were prepared for the histopathological study [62].

### 4.9. Skin Permeability Study

Franz diffusion apparatus (PermeGear, Hellertown, PA, USA) was used to conduct the ex vivo skin permeation study of the FLU–ACT-coloaded NLC gel and FLU–ACT conventional gel. The investigation was conducted on rat skin. Rats were sacrificed, and their skins were shaved and surgically removed. PBS was used to clean the skin. Prior to use, the rat skin was soaked in PBS for 30 min at 37 °C, and it was then fixed between the donor and receptor compartments with the dermal location towards the receptor chamber. The receptor chamber has a capacity of 5 mL and surface area of 0.77 cm^2^. The receptor compartment was filled with PBS (7.4) and kept at 37 °C of its original temperature. Then, 0.5 g gel was inserted in the donor chamber. Samples were taken at specified durations of 0.5, 1, 2, 3, 4,6, 12 and 24 h, and were replaced with the same volume of the buffer. The samples were analyzed with a UV–visible spectrophotometer for the quantification of the drug at 238 nm and 354 nm for FLU and ACT, respectively [63].

### 4.10. Skin Deposition Study

After the completion of the permeation study for 24 h, the quantity of the drug deposited in the skin layers was evaluated. The skin was collected after the completion of the permeation study and washed with methanol to remove any drug particles sticking to the surface of the skin. The skin was cut into small pieces to extract the deposited drug. The chopped skin was homogenized with solvent and sonicated for 1 hr. The extracted solution was centrifuged for 10–15 min, and the supernatant was collected and filtered out (using a 0.45 um filter). Finally, the filtered supernatant was analyzed for FLU and ACT by UV–visible spectroscopy [64].

#### Stability Studies

ICH guidelines (Q1A (R^2^)) were followed for conducting the stability study. The optimized FLU–ACT-coloaded NLCs were sealed in glass vials wrapped in aluminum foil and kept at two different temperatures: T_1_ = 25 ± 2 °C at 60% RH ± 5% RH (accelerated storage condition for drug substances to be stored at refrigerator temperature), and T_2_ = 40 ± 2 °C at 75% RH ± 5% RH (accelerated storage condition for general substances) for a period of 6 months. The PS, ZP, PDI and %EE of the optimized FLU–ACT-coloaded NLCs were evaluated at specified time intervals (i.e., 0, 1, 3 and 6 months) [49,65].

## Figures and Tables

**Table 1:** Optimization of FLU–ACT–coloaded NLCs.

	Factor 1	Factor 2	Factor 3	Response 1	Response 2	Response 3	Response 4
Run	Lipid	Surfactant	Drug	Particle Size	Zeta Potential	%EE ACT	%EE FLU
	(mg)	(mg)	(mg)	(nm)	(mV)	(%)	(%)
1	28	30	3	341.7 ± 2.1	−26.1 ± 1.3	68.2 ± 1.2	63.0 ± 1.6
2	32	30	4	391.4 ± 4.3	−22.0 ± 2.1	61.3 ± 1.5	56.4 ± 2.1
3	24	40	3	288.2 ± 2.3	−34.2 ± 1.0	81.6 ± 1.1	75.6 ± 1.3
4	28	40	4	323.9 ± 5.7	−27.5 ± 4.1	73.1 ± 2.1	67.8 ± 2.1
5	24	30	2	301.2 ± 4.8	−32.5 ± 3.2	77.0 ± 1.4	73.0 ± 1.8
6	28	20	4	385.1 ± 3.6	−24.2 ± 5.1	57.1 ± 1.2	52.5 ± 1.7
7	24	30	4	325.3 ± 6.1	−28.3 ± 2.5	71.8 ± 1.5	68.8 ± 1.1
8	32	40	3	348.1 ± 5.4	−26.1 ± 4.0	65.9 ± 2.4	60.0 ± 2.4
9	32	20	3	401.0 ± 3.9	−21.2 ± 2.5	56.7 ± 1.3	53.7 ± 1.4
10	28	40	2	295.8 ± 2.7	−31 ± 2.9	77.04 ± 2.0	73.2 ± 3.1
11	28	30	3	345.4 ± 8.2	−26.8 ± 4.8	69.7 ± 2.7	61.6 ± 2.2
12	28	20	2	355.7 ± 5.8	−25.9 ± 2.3	61.09 ± 2.9	57.7 ± 1.5
13	24	20	3	342.5 ± 2.3	−26.1 ± 7.2	73.8 ± 1.3	67.6 ± 1.78
14	32	30	2	360.9 ± 3.2	−25.1 ± 6.1	67.06 ± 2.5	59.08 ± 1.3

All the values represent means ± standard deviations (n = 3). EE: entrapment efficiency; nm: nanometer; mV: millivolt; mg: milligram; %: percentage.

**Table 2:** Draize scoring analysis of rat skin treated with FLU–ACT-coloaded NLC gel, 0.8% formalin and untreated.

	Time (h)	Positive Control	Negative Control	Treatment with NLCs
Erythema	1	0	3	1
	12	0	3	0
	24	0	2	0
Edema	1	0	1	0
	12	0	3	0
	24	0	2	0
PDI	1	0	4	1
	12	0	5	0
	24	0	4	0
PDII		0	4.66	0.33

PDI: primary dermal irritation; PDII: primary dermal irritation index.

**Table 3:** Drug release kinetic models of optimized FLU–ACT-coloaded NLCs.

	Zero Order		First Order		Higuchi		Hixon–Crowel		Korsmeyer–Peppas	
	R^2^	Ko	R^2^	K ^1^	R^2^	Kh	R^2^	Khc	R^2^	Kkp
FLU F	0.6977	3.516	0.9334	0.069	0.9757	14.689	0.8799	0.019	0.9770	13.889
ACT F	0.8858	2.912	0.9761	0.045	0.9484	11.685	0.9557	0.013	0.9850	7.926

**Table 4:** Skin deposition study of FLU–ACT-coloaded NLC gel and FLU–ACT conventional gel.

Drug Retained in Skin	Conventional Gel	NLC-Loaded Gel
% FLU retained in skin	4.02 ± 0.06%	21.01 ± 0.5%
% ACT retained in skin	3.13 ± 0.04%	18.89 ± 0.3%

**Table 5:** Stability study of optimized FLU–ACT-coloaded NLCs.

25 ± 2 °C at 60% RH ± 5% RH					40 ± 2 °C at 75% RH ± 5% RH			
Time (Months)	0	1	3	6	0	1	3	6
PS (nm)	288.2 ± 2.3	289.5 ± 5.4	291.2 ± 5.8	292.3 ± 1.5	288.2 ± 2.3	289.8 ± 5.5	291.1 ± 5.2	292.8 ± 3.2
ZP (mV)	−34.2 ± 1.0	−33.9 ± 2.2	−32.1.8 ± 1.8	−30.7 ± 1.0	−34.2 ± 1.0	−33.4 ± 2.0	−31.9 ± 1.5	−31.1 ± 1.7
PDI	0.345 ± 0.02	0.346 ± 0.02	0.348 ± 0.03	0.349 ± 0.04	0.345 ± 0.02	0.346 ± 0.01	0.347 ± 0.02	0.349 ± 0.05
ACT %EE	81.6 ± 1.1	79.0 ± 1.4	78.7 ± 1.5	76.1 ± 1.9	81.6 ± 1.1	78.8 ± 1.4	76.7 ± 1.7	75.9 ± 1.1
FLU %EE	75.6 ± 1.3	73.3 ± 1.1	69.9 ± 1.6	68.3 ± 1.0	75.6 ± 1.3	72.7 ± 1.2	68.8 ± 1.6	66.2 ± 1.5

## Data Availability

All data generated or analyzed during the study are included in this published article.
